# Bio-sorption for effective removal of chromium(VI) from wastewater using *Moringa stenopetala* seed powder (MSSP) and banana peel powder (BPP)

**DOI:** 10.1186/s13065-020-00724-z

**Published:** 2020-12-10

**Authors:** Tolera Seda Badessa, Esayas Wakuma, Ali Mohammed Yimer

**Affiliations:** Department of Chemistry, College of Natural Sciences, Arba Minch University, Arba Minch, Ethiopia

**Keywords:** *Moringa stenopetala*, Banana peel, Chromium(VI), FT-IR, Adsorption isotherms

## Abstract

Chromium is an extremely toxic metal in the form of Cr(VI) that causes severe environmental and health problems. Therefore, the aim of this study was to remove chromium ions from wastewater by using cost effective and environmentally friendly bio-sorbents; *Moringa stenopetala* seed powder (MSSP), and banana peel powder (BPP) and to evaluate its adsorption capacities as bio-sorbents. FT-IR characterization of the adsorbents showed that there was a change in the functional groups of the structure of both adsorbents before and after the adsorption that might be due to the adsorption processes taken place on the surface of adsorbent. Adsorption experiments were carried out as batch studies with different contact times, pH, adsorbent dose, initial metal ion concentration, and temperature. Results showed maximum removal efficiency for Cr(VI) at 120 min contact time, adsorbent dose of 20 g/L and pH 2 by MSSP and pH 4 by BPP. The percentage removal of Cr(VI) increased with increasing adsorbent dose (from 5 to 20 g/L) and contact time (from 60 to 120 min). Freundlich isotherm model showed a better fit to the equilibrium data than the Langmuir model. The kinetics of adsorption for chromium was well represented by pseudo-second order kinetic model and the calculated equilibrium sorption capacity of the model showed good agreement with the sorption capacity obtained from experimental results.

## Introduction

Chromium is a naturally occurring metal found in the environment commonly in trivalent, Cr(III), and hexavalent, Cr(VI) forms [1, 2]. The trivalent state of chromium [Cr(III)] is essential for carbohydrate metabolism in humans, whereas hexavalent chromium [Cr(VI)] is considered to be toxic [3, 4]. The hexavalent chromium is a 100-fold more toxic than trivalent form of chromium [5, 6]. This is due to the fact that it is a strong oxidizing agent that can release free radicals that can have carcinogenic effects on cells [4]. Chromium containing compounds in the form of hexavalent is used widely in different industries such as leather industry, electroplating, textile dyeing, and metal fabrication and finishing [7]. Chromium containing wastewater is one of the major pollutants of the environment. Hence, industries must treat the effluents to reduce the Chromium ions concentration in water and wastewater to acceptable levels before releasing it into the natural environment. Various conventional and advanced treatment methods have been employed for the removal of Cr ions from water and wastewater in developed countries. These include chemical precipitation, ion exchange, reverse osmosis, membrane filtration, electrodialysis, polymeric nano particles and activated carbon adsorptions [8–13]. However, these conventional and advanced technologies are expensive and non-regenerable materials used, generation of toxic sludge is often ineffective, particularly for the removal of Cr ions at low concentrations and also difficult to apply them in developing countries like Ethiopia [14–16].

Moringa is a tropical plant belonging to the family of moringaceae that grows throughout the tropical regions. *Moringa oleifera* and *Moringa stenopetala* are the two most common species among the various species of the moringa family [17, 18]. *Moringa stenopetala* is domesticated in the east African low lands and is indigenous to southern Ethiopia. *Moringa stenopetala* is often called “cabbage tree” and is an important indigenous vegetable in south Ethiopia where it is cultivated as a food crop [19, 20]. The water soluble moringa seed possess coagulating properties similar to those of synthetic cationic polymers. Moringa seeds contain cationic polypeptides with various functional groups, particularly low molecular weight amino acids. These amino acids are de-protonated to carboxylate ligands at pH range of 4 to 8 simultaneously protonating the amino group which facilitates the binding of positively charged ions with the carboxylic group [21, 22]. The use of bio-adsorbents like *Moringa stenopetala* seed powder that are easily available and effective for removal of metals could be an innovative and economical approach for treatment of industrial wastewater.

Banana is another plant most widely grown in tropical regions, cultivated over 130 countries. Ethiopia, which lies entirely in the tropics, has great potential for banana production [23]. Cavendish banana is the major fruit crop that widely grown and consumed in Ethiopia especially in the south and south western part of the country [24]. Banana is rich in polyphenols, flavonoids and dopamine which exist both in the pulp and the peel [25]. The pectin substances are complex hetero polysaccharides containing galacturonic acid, arabinose, galactose, and rhamnose as the major sugar constituents; the carboxyl groups of galacturonic acid allow pectin substances to strongly bind metal cations in aqueous solution [26]. Thus, banana peels powder could be used for the bio-adsorption of metal ions in wastewaters. The needs for cost-effective, eco-friendly and locally available alternative materials for the removal of chromium ion from aqueous solutions is essential. Therefore, *Moringa stenopetala* seed powder and banana peel powder for their adsorptive removal of Cr(VI) ions from aqueous solutions under various operating variables (contact time, solution pH, initial Cr concentration and temperature) was the focus of the current work.

## Materials and methods

### Preparation of *Moringa stenopetala* seed powders (MSSP)

**Fig. 1:**
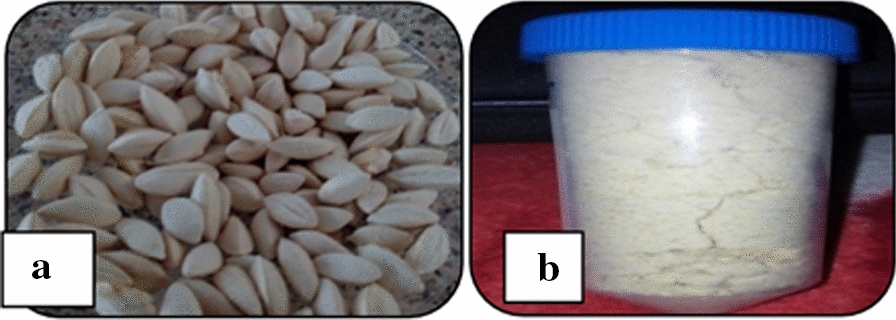
*Moringa stenopetala* sample, **a** seed and **b** seed powder

Matured and fresh seeds of *Moringa stenopetala* were collected from Arba Minch University and specimen identification of the seed was done at Addis Ababa University National Herbarium. Then the seeds were separated from their cover or shell manually. They were, air-dried and ground using a grinding mill and sieved through a mesh. Finally, the fine powder of the seed shown in Fig. 1b, was collected and kept in a clean bottle until experiments were done.

### Preparation of banana peel powders (BPP)

**Fig. 2:**
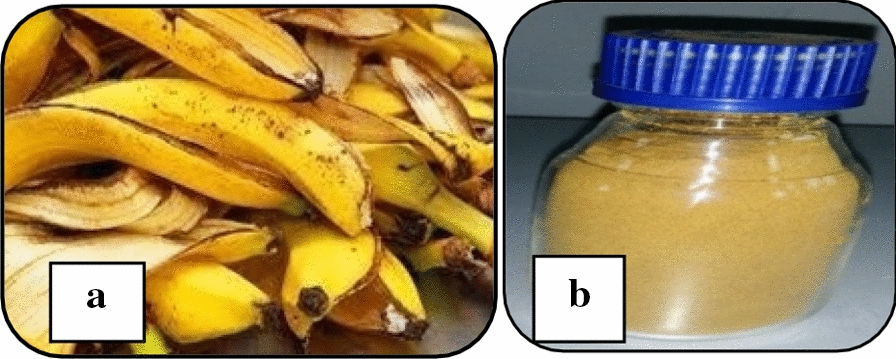
Banana peel sample **a** raw peel, **b** peel powder

The banana fruits were collected from the local market of Arba Minch City, and the peel shown in Fig. 2 was separated from the fruits and cut into smaller pieces while washing thoroughly with tap and distilled water to remove dirt particles. Finally, the wetted banana peel was air-dried, ground into a powder, and kept in a clean airtight bottle; until experiments were done.

### Preparation of adsorbate

A 1000 mg/L of Cr(VI) stock solution was prepared (2.827 g of K_2_Cr_2_O_7_ in 1000 mL volumetric flask using distilled-deionized water) [27]. Then a series of working standard solutions of different concentrations were prepared by appropriate dilution of the stock solution.

### Batch experiments

All experiments were carried out in batch mode to obtain both the thermodynamic and kinetic information. The studies were carried out at the optimized contact time, pH value, and adsorbent dosage. Before adding the adsorbent, the pH of the solution was adjusted by using 0.1 M NaOH or 0.1 M HCl solutions. To ensure equal mixing of the solution, all the experiments were performed with an orbital shaker with a constant agitation speed of 150 rpm. The equilibrium study was conducted and compared with Langmuir and Freundlich isotherm models. The kinetics experiments were studied using pseudo-first-order and pseudo-second-order models. After completing the batch experiment, the solution was separated from the adsorbent and digested in a microwave digester for the determination of its concentration using GFAAS. The blank solutions that contain the adsorbents before adsorption were also digested in the same way that the samples were done, and analysed for the chromium content The method detection limit was developed from the results to control the interferences coming from the adsorbent nature. Therefore, the result of adsorbed chromium was reported based on the method detection limit. Then, the adsorption efficiency (%) and capacity at a given contact time for the selected adsorbents were determined using Eqs. 1 and 2 [28, 29].

1 $$\% {\text{Removal}} = \frac{{\left( {{C_0} - Cf} \right)}}{{{C_0}}} \times 100,$$

2 $${\text{q}} = \frac{{\left( {{C_0} - {C_f}} \right){\text{V}}}}{m},$$

where ${C}_{0}$ is the initial concentration, $Cf$ is the concentration after adsorption, q is the metal removal in mg/g, m is the adsorbent mass in gram and V is the volume of wastewater used during the experiment.

### Adsorption isotherm

The adsorption isotherm is a graphical representation of expressing the amount of solute adsorbed per unit mass of adsorbent as a function of equilibrium concentration in bulk solution at constant temperature. The experiments were carried out under different adsorbent dose that ranged from 0.5 to 2 g with an interval of 0.5 g by keeping the previously determined parameters at the optimal level in 100 mL solution of the metal ion with an agitation speed of 150 rpm. Then, the experiments were designed and conducted for the evaluation of adsorption equilibria between the adsorbate and adsorbent and finally compared with the existing models. The results were found to be fitting both the Langmuir and Freundlich adsorption isotherm models [30–33].

The Langmuir model assumes that uptake of adsorbate molecule occurs on a homogenous surface by monolayer adsorption without any interaction between adsorbed molecules and uniform energies of adsorption [34]. The Langmuir equation is represented as:

3 $${q}_{e}=\frac{\left({q}_{m} \text{b}{C}_{e}\right)}{ (1 + \text{b}{C}_{e})}$$

where C_e_ is the equilibrium concentration of the ion (mg/L); q_e_ is the amount of ion adsorbed (mg/g); q_m_ is q_e_ for a complete monolayer (mg/g); and b is the bio-sorption equilibrium constant (L/mg).

The linear form of Langmuir isotherm model can be represented by using Eq. 4:

4 $$\frac{{{c_e}}}{{{q_e}}} = \left( {\frac{1}{{{q_m}\text{b}}}} \right) + \left( {\frac{{{c_e}}}{{{q_m}}}} \right).$$

The essential characteristics of the Langmuir isotherm can be explained by the equilibrium separation factor R_L,_ defined as:

5 $${R_L} = \frac{1}{{\left( {1 + {\text{b}}{C_o}} \right)}},$$

where b is the Langmuir constant and C_o_ is the highest metal concentration (mg/L).

Depending on the value of R_L,_ the shape of the isotherm and whether the bio-sorption is favourable or not, can be determined. The types of isotherms with R_L_ values are given in [35–37].

Freundlich isotherm model describes a multi-layer bio-sorption based on adsorption on heterogeneous surface and adsorption capacity which is related to the concentration of the adsorbent. The model can be presented as [38].

6 $${q_e} = {K_f}{C_e}^{1/\text{n}},$$

where q_e_ is the amount of ion adsorbed (mg/g); C_e_ is the equilibrium concentration (mg/L); K_f_ and 1/n are empirical constants, indicating the adsorption capacity (Freundlich constant) and adsorption intensity (which varies with the heterogeneity of the material), respectively.

The two Freundlich parameters K_f_ and 1/n can be determined graphically by plotting the experimental data and then using the Freundlich equation in the following form.

7 $$\ln {q_e} = \ln {K_f} + \frac{1}{n}\ln {C_e},$$

where K_f_ and 1/n are evaluated from the intercept and slope of the plot of lnq_e_ vs. lnC_e_. For values in the range 0.1 < 1/n < 1, bio-sorption is favourable. The greater the values of K_f_, better is the favourability of bio-sorption.

### Adsorption kinetics

The effect of time on the removal rate of Cr(VI) ion from the solution were investigated using kinetic study. Adsorption kinetics shows a large dependence on the physical and/or chemical characteristics of the adsorbent material. The experiment was conducted in a separate 250 mL Erlenmeyer flask by keeping pH, adsorbent dose and metal ion concentration at the optimum level in different time intervals (30 min, 60 min, 90 min, 120 min and 150 min) by adjusting the agitation speed at 150 rpm. Finally, the experimental data were analyzed using pseudo first-order [32] and pseudo-second order [33] kinetics models.

Pseudo-first order kinetics model was based on adsorption capacity of adsorbent and suggests that there are no interactions between ions and each ion sorbs on a local site. Therefore, the designed experiment for the bio-sorption process was undergone the pseudo-first order kinetics as the adsorption is occurring as monolayer on the surface of adsorbent and the equation is expressed as [32]:

8 $$\frac{{dq_{t} }}{{dt}} = {\text{k}}_{1} (q_{e}  - q_{t} ), $$

where q_e_ and q_t_ are the amounts (mg/g) of adsorbed pollutant on the adsorbent at equilibrium and time t; and k_1_ is the rate constant (min^−1^) of Lagergren’s first-order adsorption.

After integration and applying boundary conditions t = 0 to t = t and q_t_ = 0 to q_t_ = q_e_, the integrated form becomes:

9 $$ \log \left( {q_{e}  - q_{t} } \right) = \log q_{e}  - \frac{{k1t}}{{2.303}}. $$

The plot of log (q_e_ − q_t_) versus t gives a straight line for first-order adsorption kinetics which allows the computation of the rate constant k_1_ and q_e_ from the slop and intercept of the plot.

The pseudo-second-order kinetics model is based on the assumption that the bio-sorption follows a second order mechanism and the occupation rate of the adsorption site is proportional to the square of the number of unoccupied sites. The rate equation can be represented in the following form [33].

10 $$ \frac{{dq_{t} }}{{dt}} = k_{2} \left( {q_{e}  - q_{t} } \right)^{2} , $$

where k_2_ is the rate constant (g/mg min) of second-order adsorption.

The integrated form of the Eq. (10) becomes:

11 $$\frac{1}{{q}_{e}-{q}_{t}}= \frac{1}{{q}_{e}}+ {k}_{2}t$$

The linear form of Eq. (11) is as follows:

12 $$ \frac{t}{{q_{t} }} = \frac{1}{{k_{2} q_{e} 2}} + \frac{1}{{\left( {q_{e} } \right)t}}. $$

The k_2_ and q_e_ values of the pseudo-second-order kinetic model can be determined from the intercept and slope of the plots of t/q_t_ versus t.

### Thermodynamic study

Thermodynamic parameters provide in-depth information of inherent energetic changes that are associated with adsorption. The thermodynamic parameters such as standard Gibbs free energy change ∆G°, standard enthalpy change ∆H°, and standard entropy change ∆S° gives a better understanding of the effect of temperature on adsorption. These parameters were determined using the following equations [39].

13 $$ \Delta G^\circ  =  - RT\ln K_{c} , $$

14 $$ K_{c}  = \frac{{C_{a} }}{{C_{e} }}. $$

The Gibbs free energy change (ΔG°) is related to the entropy change (ΔS°) and enthalpy change (ΔH°) at constant temperature using Eq. 15:

15 $$ \Delta G^\circ  = \Delta H^\circ  - {\text{T}}\Delta S^\circ .$$

By combining the above two equations we get the following equation:

16 $$ \ln {\text{K}}_{{\text{c}}}  = \frac{{\Delta S^\circ }}{R} - \frac{{\Delta H}}{{RT}}, $$

where K_c_ is the equilibrium constant, T is the absolute temperature (K), R is the universal gas constant (8.314 J mol^−1^ K^−1^), ${C}_{a}$ (mg/L) is the amount of metal ion adsorbed at equilibrium, ${C}_{e}$ (mg/g) is the amount of metal ion left in the solution at equilibrium. The values of ΔH° and ΔS° were calculated from the slope and intercept of the Van’t Hoff plot of lnK_c_ versus 1/T equation [27].

## Results and discussion

### FT-IR result for BPP and MSSP

**Fig. 3:**
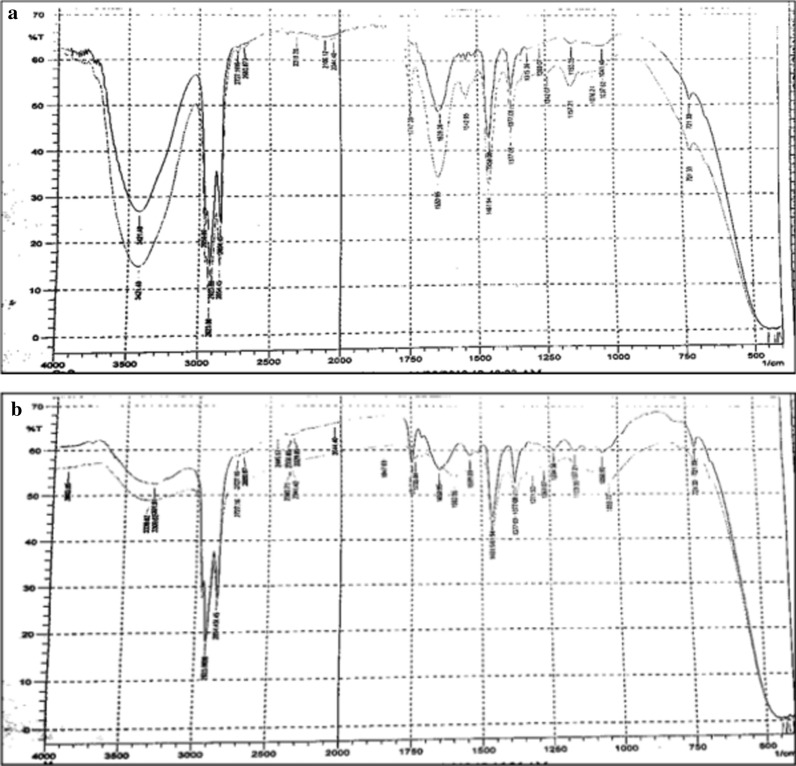
FT-IR spectra of **a** BPP and MSSP before adsorption, **b** BPP and MSSP after adsorption

The FT-IR spectra before and after adsorption of chromium were given for both BPP and MSSP in which the absorption peaks appeared in the wavenumber range of 3500 cm^−1^to 625 cm^−1^. The Fig. 3a shows the absorbance spectra of BPP and MSSP before adsorption and Fig. 3b indicates the absorbance spectra of BPP and MSSP after adsorption of chromium taken place. The spectra containing weak bands in both figures represent the absorbance spectra of BPP before and after adsorption where as the one containing stronger bands in both figures are indicating the absorbance spectra of MSSP before and after adsorption.

The FT-IR spectra in Fig. 3a shows that broad bands at 3337 cm^−1^and 3310 cm^−1^ indicate that O–H stretching vibration of carboxylic acid for both BPP and MSSP. These bands disappeared (decreased highly in intensity) in Fig. 3b, which confirms that the adsorption of chromium by both adsorbents was highly effective. In the same way a band at 1654 cm^−1^ in both figures indicate that stretching vibration of C=O group in carboxylic acid. In Fig. 3b these bands for both BPP and MSSP were also decreased in intensity that confirms adsorption of chromium on both adsorbents.

### Effect of contact time

**Fig. 4:**
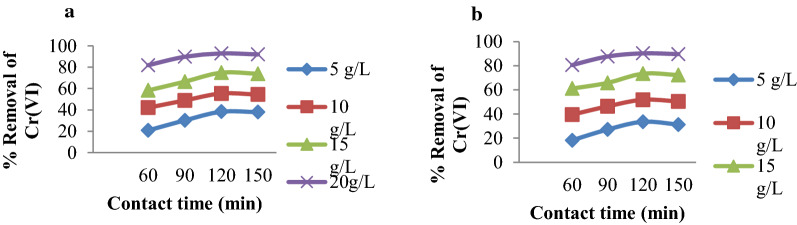
Effect of contact time on % removal of: A/Cr(VI) by MSSP and B/Cr(VI) by BPP (concentration 30 mg/L, contact time 60, 90, 120 and 150 min, adsorbent dose 5, 10, 15 and 20 g/L solution, agitation speed 150 rpm and pH = 2 for MSSP, pH = 4 for BPP)

The percentage removal of Cr(VI) ions and the adsorption capacity of MSSP and BPP as a function of contact time was conducted at 60, 90, 120 and 150 min using different pH and dose at a constant initial concentration, agitation speed and temperature. The experimental results shown in Fig. 4 indicate that the percentage removal of Cr(VI) ion increases with increasing contact time up to 120 min. Further increase in contact time did not make any change in removal and adsorption capacity. Higher removal efficiency and adsorption capacity was observed at 120 min for both the adsorbents. Hence, 120 min was chosen as the equilibrium time. Because at the beginning of the process, adsorption is fast due to the availability of large active binding site but as time has gone, the process slow down as active binding sites are filled by the metal ion.

### Effect of pH

Solution pH is one of the most important parameters while assessing the adsorption capacity of an adsorbent for metal ion sequestration from aqueous. The pH of the system controls the adsorption capacity due to its influence on the surface properties of the adsorbent and ionic forms of the chromium solutions.

Cr(VI) adsorption was studied as a function of pH over a range of 2–8 for MSSP and BPP at an initial concentration of 30 mg/L wastewater. The results of the experiment shown in Fig. 3 indicate that optimal Cr(VI) removal efficiency of MSSP and BPP were obtained at pH 2.0 and pH4.0 respectively for 120 min contact time. It is evident that chromium(VI) removal efficiency increases with decrease in pH [31]. The favorable effect at low pH might be due to the neutralization of negative changes on the surface of the adsorbents by excess hydrogen ions, thereby facilitating the diffusion of the hydrogen chromate ion (HCrO_4_^−^) and its subsequent adsorption, because HCrO_4_^−^ is the dominant anionic form of Cr(VI) between pH 1.0 and 4.0. This ionic form was found to be preferentially adsorbed on the surface of the adsorbent. The possible explanation for higher adsorption in the acidic region is that the Cr_2_O_7_^2−^ ion is oxidized to Cr_3_^+^. Being small in size, it can be easily replaced by the positively charged species The following reaction mechanism might be taken place during adsorption processes on the surface of adsorbents [40, 41].

$$2{\text{H}}^{ + }  + 2{\text{HCrO}}_{4}^{ - } \leftrightarrow 2{\text{H}}_{2} {\text{CrO}}_{4} \leftrightarrow 2{\text{H}}_{2} {\text{O}} + {\text{Cr}}_{2} {\text{O}}_{7}^{{2 - }}  + 2{\text{H}}^{ + }  $$

$$2{\text{H}}_{2} {\text{O}} + {\text{Cr}}_{2} {\text{O}}_{7}^{{2 - }}  + 2{\text{H}}^{ + }  \leftrightarrow 2{\text{H}}_{2} {\text{Cr}}_{4}  \leftrightarrow 2{\text{CrO}}_{3}  + 2{\text{H}}_{2} {\text{O}} $$

**Fig. 5:**
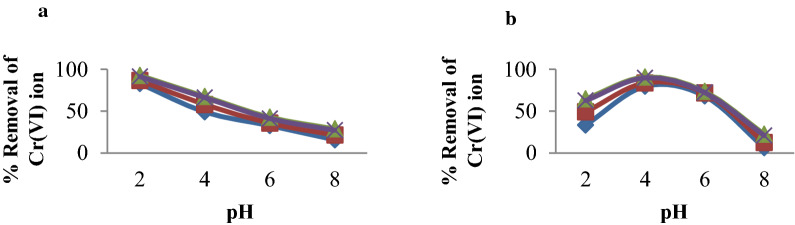
Effect of solution pH on % removal of: **a** Cr(VI) by MSSP and **b** Cr(VI) by BPP (concentration 30 mg/L, pH, 2, 4, 6 and 8 and contact time 60, 90, 120 and 150 min, agitation speed 150 rpm)

Therefore, chromium removal in mg/g by MSSP and BPP increases with increasing contact time from 60 to 120 min. for all pH values; this is because the solution pH is directly related to the large availability of positively charged active sites on the surface of the adsorbent to bind with the metal ions as the solution pH decreases, the surface of the adsorbents exhibits increasing positively charged active sites. As pH decreases, the removal efficiency becomes increasing that was shown in Fig. 5. The maximum adsorption of Cr(VI) was observed at the acidic range because, at lower pH, there is an increase in H^+^ ions on the adsorbent surface, and the presence of HCrO_4_^−^ ions result in significantly strong electrostatic attraction [42].

### Effect of adsorbent dose

**Fig. 6:**
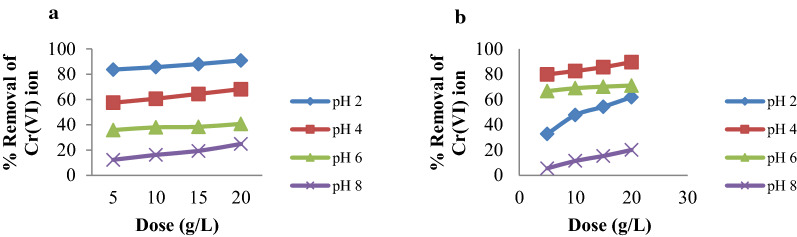
Effect of adsorbent dose on % removal of: **a** Cr(VI) by MSSP and **b** Cr(VI) by BPP (concentration 30 mg/L, agitation speed rpm, adsorbent dose 5, 10, 15 and 20 g/L solution, pH 2, 4, 6 and 8)

The effect of the adsorbent dose on the removal of Cr(VI) ions onto MSSP and BPP was conducted by using 5, 10, 15 and 20 g/L of adsorbent dose under variable pH and contact time on keeping agitation speed and initial concentration constant. Figure 6 shows that the percent removal of chromium by MSSP increases from 24.85 to 90.96% and by using BPP it ranges from 20.06 to 89.62% with an increase in adsorbent dose from 5 to 20 g/L at all pH values. The increase in adsorbent dose generally increases the percent removal of the metal ion due to increased surface area of the adsorbent which increases the number of available binding sites for the adsorption process. On the other hand, the quantity of adsorbate metal ion per unit weight of the adsorbent decreases with increasing in the adsorbent dose and this may be due to high adsorbent concentration. If the available metal ion to be sorbed is not sufficient to completely cover the available active sites of the adsorbents, it is leading to the reduction of metal ion uptake [43].

On the other hand, adsorption capacity in mg/g decreases with increasing adsorbent dose for all pH values. The highest removal efficiency was observed at 5 g/L of adsorbent. Adsorbent dose has also its own effect on the removal efficiency at various contact time. Higher adsorption was obtained at 20 g/L for all contact times but at this dose percent removal variations among different contact times were small. This may be explained by the presence of screening effect when the adsorbent dose increases [44]. Thus, the removal efficiencies of chromium ions at optimal adsorbent concentration were selected as 20 g/L [45].

### Effect of initial concentration of chromium

**Fig. 7:**
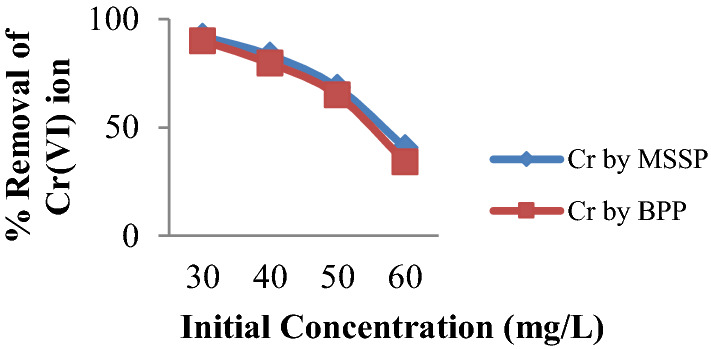
Effect of initial concentration on % removal of Cr(VI) by MSSP and by BPP [dose 20 g/L, contact time 120 min and pH 2 for Cr(VI) by MSSP and pH 4 for Cr(VI) by BPP]

The experiment was carried out under optimal conditions and different initial concentration ranging from 30 mg/L up to 60 mg/L with an interval of 10 mg/L. As shown in Fig. 7, the increasing of the initial concentration of chromium increases adsorption capacity of the adsorbents but decreases percentage removal of the metal. Maximum % removal of chromium by MSSP is 92.17% and by BPP is 90.07 at 30 mg/L. The decrease in percentage removal of the metal ion with increasing initial concentration may be due to the saturation of adsorption sites on the adsorbent surface. This can be explained by the fact that, at low concentration, the metal ions interact with the binding sites and result in maximum adsorption. This is because, at low concentration, the ratio of available surface to the initial metal ion concentration is larger, so the removal is higher [46]. On the other hand, maximum Cr(VI) ion adsorption capacity of MSSP is 1.71 mg/g and that of BPP is 1.63 mg/g at initial concentration of 50 mg/L. The increase in adsorption capacity with increasing initial concentration is due to the fact that, at fixed adsorbent dose with increasing the metal concentration, all the available active sites of the adsorbent would be fully exposed to get occupied by the metal ions that are in excess saturating and yielding a higher adsorption capacity [47].

### Effect of temperature

**Fig. 8:**
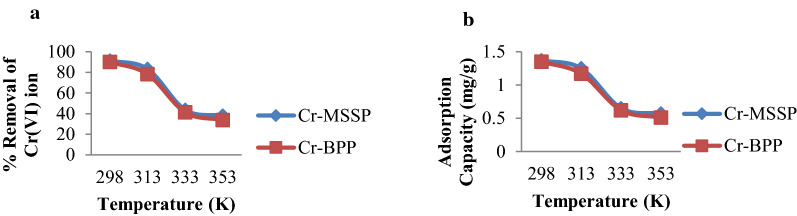
Effect of temperature on: **a** % removal of Cr(VI) and **b** adsorption capacity of MSSP [concentration 30 mg/L, dose 20 g/L, contact time 120 min. and pH 2 for Cr(VI) by MSSP and pH 4 for Cr(VI) by BPP]

The effect of temperature on adsorption capacity of Cr(VI) onto MSSP and BPP was studied at a temperatures of 293 k, 313 k, 333 k and 353 k at the optimal conditions of the solution. As shown in Fig. 8, an increase in temperature from 293 to 353 k decreases the percentage removal from 91.40 to 37.93% that contains MSSP and from 90.03 to 33.80% for chromium solution that contains BPP respectively. Similar to percent removal, adsorption capacities of MSSP and BPP on the adsorption of Cr(VI) ion decreased from 1.37 to 0.57 mg/g and 1.35 mg/g to 0.51 mg/g with increasing temperature from 293 to 353 k. This fall of metal uptake capacity of the adsorbents during the rise in temperature might be due to desorption caused by an increase in the available thermal energy. Higher temperature induces higher mobility of the adsorbate causing desorption or the damage of active binding sites in the biomass [48]. The decrease in adsorption capacity with increasing temperature indicates that adsorption process is exothermic in nature.

### Adsorption isotherm

**Fig. 9:**
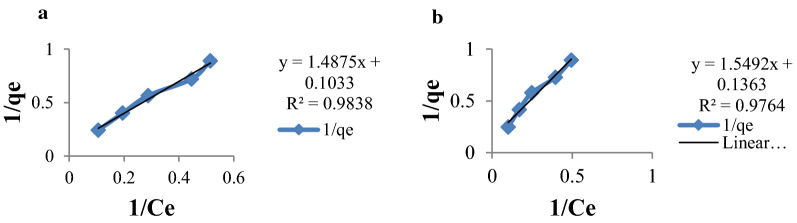
Langmuir adsorption isotherms for: **a** chromium by MSSP and **b** chromium by BPP

The Langmuir plot between 1/q_e_ vs. 1/C_e_ for the sorption of Cr(VI) ion by MSSP and BPP are drawn in Fig. 9. It was found that the values of maximum adsorption capacity of MSSP and BPP were 9.709 mg/g and 7.353 mg/g, respectively (Table 1). The essential characteristics of the Langmuir isotherm can be explained by the equilibrium separation factor R_L_. The R_L_ values for sorption of Cr(VI) ion by MSSP and BPP in this study were 0.325 and 0.275, respectively. This indicates that MSSP and BPP are favourable adsorbents for the removal of chromium.

**Table 1:** Parameters of Langmuir and Freundlich adsorption isotherms of chromium

Metal ion	Langmuir isotherm				Freundlich isotherm		
	q_m_	b	R_L_	R^2^	K_f_	1/n	R^2^
Cr(VI)-MSSP	9.709	0.0692	0.325	0.983	0.685	0.792	0.993
Cr(VI)-BPP	7.353	0.0879	0.275	0.976	0.635	0.779	0.983

**Fig. 10:**
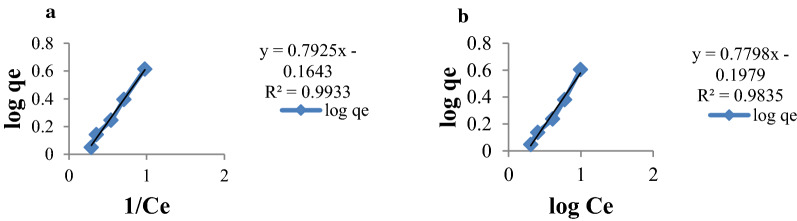
Freundlich adsorption isotherms for: **a** chromium by MSSP and **b** chromium by BPP

The Freundlich adsorption model is based on adsorption on heterogeneous surface and adsorption capacity related to the concentration of the adsorbent. The value of Freundlich constant 1/n and K_f_ for the sorption of Cr(VI) by MSSP and BPP were obtained from the slope and intercept of the plot logq_e_ vs. logC_e_. K_f_ which is an indicator of adsorption capacity of adsorbent and the higher the adsorption capacity, the higher the value of K_f_. On the other hand, 1/n is a measure of intensity of adsorption. Therefore, the K_f_ value for the sorption of Cr(VI) ion by MSSP and BPP are 0.685 and 0.635 whereas the 1/n values for the sorption of Cr(VI) are 0.792 and 0.779 respectively. This indicates that both MSSP and BPP have high chromium sorption capacity and are favourable adsorbents from wastewater. The values of correlation coefficients (R^2^) of the Freundlich model given in Fig. 10, which were 0.993 and 0.983 for MSSP and BPP respectively and these values are larger than the R^2^ values of the Langmuir model (0.983 and 0.976). This indicates the Freundlich model fits better to the adsorption data and thus it is more suitable to be used to describe the relationship between the amounts of Cr(IV) ion adsorbed by MSSP and BPP.

### Adsorption kinetics

**Fig. 11:**
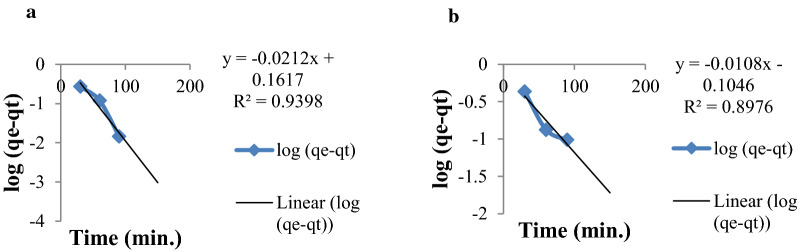
Pseudo-first order kinetics plot for **a** chromium by MSSP and **b** chromium by BPP

**Fig. 12:**
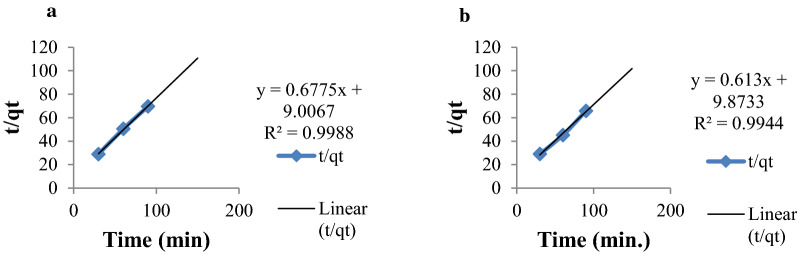
Pseudo-second order kinetics plot for **a** chromium by MSSP and **b** chromium by BPP

From the experimental data shown in Table 2, the calculated and experimental equilibrium adsorption capacity, q_e_(cal) and q_e_(exp), of pseudo-second-order kinetic model was close to each other than the pseudo-first-order kinetic model. This indicates that the pseudo-second order kinetics is the best kinetic model that fits to the experimental data for sorption of Cr(VI) by MSSP and BPP. The correlation coefficients (R^2^) of pseudo-second-order kinetic model of chromium by MSSP and BPP (0.998 and 0.994) are closer to unity compared to the pseudo-first-order kinetic model (0.939 and 0.897). This indicates pseudo-second-order kinetic model is better model to the kinetics of adsorption onto the adsorbents it is indicated in Figs. 11 and 12.

**Table 2:** Pseudo-first and second order constants for chromium by MSSP and BPP

Metal ion	q_exp_	Pseudo first order kinetic model			Pseudo second order kinetic model		
		q_cal_ (mg/g)	k_1_ (min^−1^)	R^2^	q_cal_(mg/g)	k_2_ (g/mgmin)	R^2^
Cr-MSSP	1.308	1.449	0.0484	0.939	1.477	0.051	0.998
Cr-BPP	1.4645	0.7870	0.023	0.897	1.631	0.038	0.994

### Thermodynamics study

The values of the thermodynamic parameters for adsorption of Cr(VI) ion onto MSSP and BPP are shown in Table 3. The increase in temperature made the values of ΔG° more positive. A negative value of Gibbs free energy change (ΔG°) confirms the feasibility of the process and spontaneous nature of the sorption of Cr(VI) ion onto MSSP and BPP. The change in Gibbs free energy decreases with increasing temperature which indicates that a lower temperature favours adsorption.

**Table 3:** Thermodynamicparameters for Cr(VI) ion adsorptionon to MSSP and BPP atdifferent temperatures

T (K)	Cr-MSSP				Cr-BPP			
	K°	∆G° (kJ/mol)	ΔH° (kJ/mol)	ΔS° (kJ mol^−1^)	K_c_	∆G° (kJ/mol)	ΔH° (kJ/mol)	ΔS° (kJ mol^−1^)
293	1.88	− 1.535	39.28	0.129	2.21	− 1.924	42.56	0.140
313	4.02	− 3.617			5.57	− 4.476		
333	26.05	− 9.026			28.45	− 9.275		
353	32.67	− 10.243			38.94	− 10.742		

The adsorption process involves energy changes that could result in a positive or negative value of ΔH°. The negative values of ΔH° in this study indicates adsorption of Cr(VI) ion onto MSSP and BPP is exothermic in nature. If enthalpy change, (ΔH°) of an adsorbent is higher than 40 kJ mol^−1^, the process is said to be chemisorption which includes strong electrostatic chemical bonding between the metal ions and adsorbent surface. On the other hand, enthalpy change less than 20 kJ mol^−1^ indicate the physical nature of the adsorption process. In this study, all the values of ΔH° are greater than 40 kJ mol^−1^ revealing that chemisorption was responsible for the adsorption Cr(VI) ion onto MSSP and BPP. The positive values of entropy, (ΔS°), indicate the increase in randomness of sorption processes of Cr(VI) ion at the solid-liquid interface of MSSP and BPP.

## Conclusions

From the current study, it can be concluded that the seed powders of *Moringa stenopetala* and banana peels can be used to remove Cr(VI) ion from wastewater. FT-IR characterization of the adsorbents showed that there was a change in the functional groups on the structure of both adsorbents before and after the adsorption processes. Percentage removal of Cr(VI) ion increases with decreasing pH, increasing adsorbent dose and contact time. Adsorption isotherm were described using the Langmuir and Freundlich models. The Freundlich model fits better to the adsorption data. The kinetics study reveals that the sorption of Cr(VI) ions are faster in the pseudo-second order kinetics than pseudo first order kinetics. Thermodynamic study was investigated using ΔG°, ΔH° and ΔS° and the negative values of ΔG° shows the spontaneous nature of the sorption process and the positive value s of ΔS° indicates the randomness of the adsorption processes. The use of the greener bio-sorption process using MSSP and BPP adsorbents is important for the removal of Cr(VI) ions from wastewater in order yo safe the environment. When comparing the two adsorbents, the removal efficiency of MSSP is better than BPP.

## Data Availability

The authors declare that the manuscript contains the minimal dataset that is required to interpret, replicate, and build upon the methods and findings reported in the article. Raw data can be shared via correspondence upon reasonable request.

## Abbreviations

MSSP: *Moringa stenopetala* seed powder
BPP: Banana peel powder
FT-IR: Fourier transformer infrared
∆*G*°: Gibbs free energy
∆*H*°: Enthalpy change
∆*S*°: Entropy change
K: Equilibrium constant
k: Rate constant
q_e_: The amount of ions adsorbed (mg/g)
Q_m_: The amount of ion adsorbed on monolayer (mg/g)
C_0_: Initial concentration of the ion (mg/L)
C_e_: Concentration of ion at equilibrium (mg/L)
R: Universal gas constant (8.314 JK^−1^ mol^−1^)
